# Anakinra for Recurrent Fevers in Systemic Lupus Erythematosus

**DOI:** 10.7759/cureus.3782

**Published:** 2018-12-27

**Authors:** Eric Dein, Ashley Ingolia, Caoilfhionn Connolly, Rebecca Manno, Homa Timlin

**Affiliations:** 1 Rheumatology, Johns Hopkins Bayview Medical Center, Baltimore, USA; 2 Internal Medicine, North Oaks Health System, Hammond, USA; 3 Internal Medicine, The Johns Hopkins University School of Medicine, Baltimore, USA; 4 Rheumatology, The Johns Hopkins University School of Medicine, Baltimore, USA

**Keywords:** fever of unkown origin, lupus, anakinra

## Abstract

Fever is a common manifestation in systemic lupus erythematosus (SLE) and may be associated with disease activity, but should be closely evaluated for infection, drug reaction, thromboembolism, malignancy, or other etiology. We present the case of a 44-year-old Peruvian female with SLE with periodic high fevers and elevated high-sensitivity C-reactive protein (hs-CRP) levels, treated with anakinra, an interleukin-1 (IL-1) inhibitor. Following the birth of her first child, she developed frequent episodic fevers followed by multiple hospitalizations, approximately two to three times per year. She was started on anakinra in September 2016 and had improvement of fevers and joint symptoms. On 26-month follow-up, she had one episode of fever with bandemia requiring hospitalization but otherwise remained afebrile with a significant drop in CRP. Anakinra is well-tolerable and safe due to a short half-life. We report that the inhibition of IL-1 may be a safe and effective treatment for recurrent fevers in SLE.

## Introduction

Systemic lupus erythematosus (SLE) is a multisystem disease with a variety of different clinical symptoms. Fever is a common manifestation in SLE, seen in 36% to 86% of patients [1-2]. Lupus-associated fevers may be related to disease activity, but should be closely evaluated for infection, drug reaction, thromboembolism, malignancy, or other etiology. Fever is the main cause of hospital admissions and is a marker of disease activity on the Modified Systemic Lupus Erythematosus Disease Activity Index (M-SLEDAI) [1-2].

## Case presentation

We present the case of a 44-year-old Peruvian female with SLE with periodic high fevers and elevated C-reactive protein (CRP) levels, treated with anakinra.

In 2003, at the age of 29, she presented with rash, headache, intermittent joint swelling, and recurrent fevers. The following year, she was diagnosed with aseptic meningitis, pneumonitis, and possible adult Still’s disease. She was lost to follow-up over the next six years, though was started on hydroxychloroquine by an outside provider during this time. In 2010, she returned for follow-up during pregnancy and the diagnosis of systemic lupus erythematosus was made. Her lupus was characterized by a positive anti-nuclear antibody ([ANA], 1:160 H/S), elevated levels of anti-beta 2 glycoprotein antibody (26-36, normal 0-20 Std IgM units), positive lupus anticoagulant by dilute Russell viper venom time (dRVVT) confirmation, low complement 3 (C3), leukopenia, thrombocytopenia (<100, normal 150-350 K/mm^3^), fevers, alopecia, and arthritis. In April 2011, further workup revealed low-titer positive anti-smooth muscle antibodies and elevated liver function test (LFT) results and was diagnosed with autoimmune hepatitis. Transaminitis improved on prednisone and azathioprine and later switched to mycophenolate mofetil.

Beginning in 2010, the patient had recurrent episodic fevers and multiple hospitalizations. Her fevers occurred throughout the day without a specific pattern with a temperature of 102-104 °F. The fevers were associated with markedly elevated levels of high-sensitivity CRP (hs-CRP), with the highest hs-CRP of 281 mg/l (normal <0.29). She was hospitalized two to three times per year (mostly in spring and fall), with extensive infectious work-ups, as well as a negative periodic fever panel.

In September 2016, she was started on anakinra, an interleukin-1 (IL-1) inhibitor. On the current regimen of anakinra 100 mg daily, mycophenolate mofetil 500 mg twice daily, hydroxychloroquine 200 mg twice daily, and a tapering dose of prednisone (<5 mg), she had improvement of fevers and joint symptoms. On a 26-month follow-up since anakinra initiation, she has had one episode of fever but otherwise remained afebrile with a significant drop in hs-CRP levels. During the febrile episode in July 2018, she had a fever (103 °F) with bandemia of 22% (normal 2% to 6%) and elevated hs-CRP levels of 96.4 (normal <0.29). Infectious workup was unrevealing and was restarted on anakinra after discharge.

## Discussion

Patients with SLE are at high risk of fever, given the immune system dysfunction and immunosuppressive medications. Therefore, a fever in SLE should always be first considered for infectious causes [3]. Rovin et al. defined three criteria for evaluating fevers in SLE and determining if it is related to disease activity, rather than an infectious etiology [4]. First, an absence of infection should be demonstrated through extensive bacteriological testing. Next, the patient should demonstrate signs and symptoms of ongoing lupus activity in conjunction with the fever. Finally, upon escalation of immunosuppression, physicians should closely evaluate for evidence of infection. SLE fever can, therefore, be diagnosed in a patient with ongoing lupus disease activity after an extensive infectious workup and careful monitoring with immunosuppression. Physicians should also be mindful of other non-infectious etiologies of fever including drug reaction, thromboembolism, malignancy, and vasculitis [1]. Physicians treating patients with SLE should maintain a broad differential diagnosis, and a careful history taking, medication reconciliation, physical examination, and laboratory testing are essential in the evaluation.

Temperature regulation is controlled by the thermoregulatory center of the anterior hypothalamus and responds to pyrogenic cytokines that induce a fever response. These cytokines include IL-1, tumor necrosis factor (TNF), interleukin-6 (IL-6), interferon alpha (IFN-α), interferon beta (IFN-β), and interferon gamma (IFN-γ). They are produced by activated macrophages and monocytes in an inflammatory response to modulate the body temperature [5]. In particular, a meta-analysis revealed that polymorphisms of IL-1 have been associated with susceptibility to SLE in the European and Asian populations [6].

Anakinra is a recombinant human IL-1 receptor antagonist (rHulL-1Ra) used in the treatment of rheumatoid arthritis and auto-inflammatory conditions. It is well-tolerated with a favorable safety profile and a short half-life [7-8]. Ostendorf et al. studied the safety and tolerability of anakinra in four patients with lupus arthritis. They found that IL-1 was safe, well tolerated, and led to clinical and serological improvement in their small study of IL-1 inhibition in patients with SLE [9].

## Conclusions

Fevers are a common manifestation of SLE but all patients should be thoroughly evaluated for infectious and alternative etiologies of fever. In patients with recurrent fevers secondary to disease activity, IL-1 inhibition with anakinra may be a safe and effective treatment.
